# Effects of Activity Tracker-Based Counselling and Live-Web Exercise on Breast Cancer Survivors during Italy COVID-19 Lockdown

**DOI:** 10.3390/jfmk6020050

**Published:** 2021-06-09

**Authors:** Andrea Di Blasio, Teresa Morano, Federica Lancia, Gianluca Viscioni, Angelo Di Iorio, Simona Grossi, Ettore Cianchetti, Lucia Cugusi, Stefano Gobbo, Marco Bergamin, Anna D’Eugenio, Laura Masini, Massimo Rinaldi, Maria Teresa Scognamiglio, Anastasios Vamvakis, Giorgio Napolitano

**Affiliations:** 1 Department of Medicine and Ageing Sciences, “G. d’Annunzio” University of Chieti-Pescara, Via Polacchi L. 11, 66100 Chieti, Italy; moranoteresa@gmail.com (T.M.); lanciafederica@gmail.com (F.L.); gianluca.viscioni@gmail.com (G.V.); a.diiorio@unich.it (A.D.I.); annadeugenio.medicinaintegrata@gmail.com (A.D.); lauramasini.medicinaintegrata@gmail.com (L.M.); drmassimorinaldi@gmail.com (M.R.); giorgio.napolitano@unich.it (G.N.); 2 Eusoma Breast Centre, “G. Bernabeo” Hospital, ASL02 Lanciano-Vasto-Chieti, c.da S. Liberata, 66026 Ortona, Italy; sgrossi@unich.it (S.G.); ettore.cianchetti@gmail.com (E.C.); mt.scognamiglio66@gmail.com (M.T.S.); 3 Department of Biomedical Sciences, University of Sassari, Viale San Pietro 43/B, 07100 Sassari, Italy; lucia.cugusi@uniss.it; 4 Department of Medicine, University of Padova, Via Giustiniani 2, 35128 Padova, Italy; stefano.gobbo@unipd.it (S.G.); marco.bergamin@unipd.it (M.B.); 5 Department of Internal Medicine, Papageorgiou Hospital, Aristotle University of Thessaloniki, 56403 Thessaloniki, Greece; tvamvakis@yahoo.gr

**Keywords:** light-intensity physical activity, Polar Loop 2, sedentary time, breast cancer

## Abstract

Background: To prevent and fight the increase of daily sedentary time and to promote and stimulate the positive effects of physical activity and exercise on health, both traditional interventions and new strategies are important for breast cancer survivors (BCS). The research goal was to compare the effects of weekly personal feedback, based on objectively measured physical activity, on the trends of both daily sedentary time and on the physical activity of BCS (E^−^ group) with those of an intervention also including online supervised physical exercise sessions (E^+^ group), during the Italy COVID-19 lockdown. Methods: The Italian COVID-19 emergency allowed the possibility to also observe the effects of social and personal limitations. A total of 51 BCS were studied over an 18-week period and had an objective registration of day-to-day sedentary time, physical activity, and sleep. Both subsamples received weekly or fortnight personal feedback. Data were analysed considering four key periods, according to the COVID-19 emergency steps. Results: Statistical analysis showed an additive effect for sedentary time and a multiplicative effect both for light-to vigorous and light-intensity physical activities. The E^−^ group had a high overall sedentary time and a different trend of light-to vigorous and light-intensity physical activities, with a reduction from the 1st to the 2nd periods (national and personal restrictions), showing a significant rise just at the end of the national restrictions. Conclusions: The use of an activity tracker and its accompanying app, with the reception of weekly tailored advice and supervised online physical exercise sessions, can elicit proper physical activity recomposition in BCS in the COVID-19 era.

## 1. Introduction

A diagnosis of cancer and both pharmacological and non-pharmacological treatments of breast cancer could have negative effects on daily physical activity (reducing it), while sedentary time is increased [1,2,3,4,5,6]. Indeed, according to a study by De Groef et al. [5], 2 years after surgery, all activity domains were still significantly lower compared to preoperative values. After the first 12 months, the only significant improvement was seen in the occupational domain, while Gal et al. [6] found that breast cancer survivors, with and without systemic treatment, were less likely to spend time in physical activity compared to the general population, until 3 years post-diagnosis. The contemporaneous increase of sedentary time and reduction of physical activity needs particular and early attention due to its negative consequences on psychophysical health [7], as well as through its characteristic pro-inflammatory pattern, which is considered the starting point of the most common chronic non-communicable diseases [8], including breast cancer onset and recurrence. To prevent and fight the increase of daily sedentary time and to promote and stimulate the positive effects of physical activity and exercise on health [9,10], both traditional interventions, based on in-person ambulatory counselling and supervised adapted physical exercise sessions [9,10,11,12], and new strategies are important to reach as many women as possible, according to personal differences that are linked with psychological, familiar, working, and environmental differences. Thanks to advances in technology, increasing literature supports the importance of the use of activity trackers to improve the daily physical activity of breast cancer survivors, both alone [13,14,15,16,17] and integrated into supervised exercise programmes [18]. Indeed, they can stimulate people to be more physically active and less sedentary, as they provide insights into physical activity variables promoting “self-knowledge, and health, through numbers” [19]. Therefore, the study by Wu et al. [18] furnished important results, underlying the need to combine technology with both human feedback and interventions. Indeed, breast cancer survivors participating in a combined 12-week in-person physical exercise programme and physical activity promotion, using activity trackers, underlined that the use of an activity tracker and its accompanying app raised lifestyle awareness. Therefore, patients need personalized advice and a more realistic representation of total daily physical activity, together with more integration between the interventions concerning their recovery. This is of particular importance in the COVID-19 era. Worldwide, during the COVID-19 pandemic lockdown, the "stay-at-home" measures adopted to counteract the spread of the virus would dramatically reduce the physical activity levels of the general population [20,21], including cancer patients, suffering from psychophysical constriction characterized by the contemporaneous increase of sedentary time and reduction of daily physical activity, while the fear of the virus increases, together with psychosocial and emotional disorders, sleep disruption, and consequently sedentary time [22,23,24,25,26,27]. The described situation is particularly dangerous for cancer survivors as leads to poor psychophysical health, due to the fact that poor physical activity is linked to some of the side effects of cancer treatments, such as poor sleep, reinforcing the negative loop [28,29]. To prevent and counteract the negative consequences of the “stay-at-home” measures it was widely suggested to support personalized and supervised physical activity programs, with the option to group-play physical activity programs (e.g., exergames) [25]

Therefore, in order to optimize the recovery of breast cancer survivors, the original research goal was to compare the effects of weekly personal feedback, based on objectively measured physical activity, on daily sedentary time, and on physical activity of breast cancer survivors with those of an intervention also including online supervised physical exercise sessions. In this case, the consequences of the COVID-19 emergency (i.e., the government restrictions to counteract the spread of the virus) occurred during the execution of the study, allowing us to also observe the interaction of the treatments with the phases of the first Italian lockdown. Therefore, the final research goal was to verify whether merging weekly personalized feedback and online supervised physical exercise sessions confers major benefits on daily sedentary time and on physical activity of breast cancer survivors than just weekly personalized feedback, in the presence of a personal confinement and of its progressive regression. Our hypothesis was that the absence of supervised workouts, even in the presence of tailored personal suggestions, has lower power in the maintenance/improvement of daily sedentary time and physical activity characteristics of breast cancer survivors, in the presence of a personal confinement and, also, during its progressive regression.

## 2. Materials and Methods

### 2.1. Participants

The Integrative Medicine Clinic of both ASL02 of Lanciano-Vasto-Chieti (Italy) and Department of Medicine and Ageing Sciences of the “G. d’Annunzio” University of Chieti-Pescara (Italy) at “G. Bernabeo” Hospital (Ortona, Italy) recruited study participants. A total of 51 breast cancer survivors (50.98 ± 6.28 years), among those who had visits from 1 October 2019 to 12 January 2020, matching both inclusion and exclusion criteria were selected for this study. The inclusion criteria for this study were age between 30–60 years, 6–48 months after breast surgery, actual hormone therapy, and participation in the “Angel Project”, which is described in the following section. The exclusion criteria for this study were actual chemotherapy, actual radiotherapy, actual diseases limiting motion, actual chronic use of hypnotic pills, actual pharmacological treatment for anxiety and/or depression or no interest in participating in live online physical exercise sessions. The term “actual” relates to a period starting from the date of the basal evaluation of each participant and continuing until the end of the study. The Ethics Committee of Chieti-Pescara approved this study (# 312/2015), and participants gave their written informed consent.

### 2.2. Study Design

As displayed in Figure S1, the Integrative Medicine Clinic, which was activated on 15 November 2017, furnishes integrative support for breast cancer survivors during the follow-up phase, including evaluations, behavioural counselling, and interventions. The clinic’s role was significant in directing patients regarding physical activity, sleep, body composition and nutrition; acupuncture; analysis and control of blood, salivary, metabolic, immune and endocrine parameters; psychotherapy, mindfulness, and both adapted and supervised physical exercise. Patients participating in the “Angel Project” were requested to continuously wear a scientifically validated commercial accelerometer (i.e., Polar Loop 2 (Kempele, Finland)) [30,31,32] to record and remotely control daily physical activity, sedentary time, and sleep characteristics through the use of a dedicated website (i.e., Polar Flow (Kempele, Finald)), to receive personalized weekly feedback from the Integrative Medicine Clinic for 18 consecutive weeks. In the same period, they received personalized qualitative dietary suggestions, with a fortnight frequency. As the objective of the project was to educate persons to progressively self-evaluate their lifestyle (i.e., nutrition, daily physical activity, sedentary time, and sleep characteristics) during the first 18 weeks, as well as for an additional 12 weeks, each participant in the project had their data from the past week sent to the Integrative Medicine Clinic for interpretation and feedback, after having uploaded the activity tracker’s data to the website. The feedback, which was inherent to sedentary time, physical activity, and sleep characteristics, listed their positive and negative points and how the latter needed to be improved in order to receive feedback concerning self-interpretation. With a fortnight frequency, each participant did the same, with feedback concerning the qualitative characteristics of their nutrition and receiving the feedback about their appropriateness. During the whole experimental period, each participant was followed by a different researcher in each field, remaining the same until the end of the 30-week period. Each researcher followed a maximum of 30 persons. In each field, participants were randomly assigned to one of two available researchers during the recruitment process (in an alternate manner). In each field, researchers had the same cultural background and formation, and to properly set their own work, they had the possibility to see the interaction of the assigned participant with the other researcher.

All selected participants started the project in the same day, 3 February 2020, and finished the first phase on 8 June 2020. Due to the COVID-19 emergency, which was characterized by a national quarantine in Italy from 8 March 2020 to 4 May 2020, the first phase of observation period had the following characteristics: (i) From 3 February 2020 to 8 March 2020 the lifestyles of participants were not influenced by government restrictions; (ii) from 8 March 2020 to 4 May 2020 the “stay-at-home” measures, which were nationally adopted to counteract the spread of the virus, dramatically influenced the lifestyles of participants, dramatically restricting the possibility to go outside the home, work, and provide for primary necessities; (iii) from 4 May 2020 to 1 June 2020, due to the progressive reduction of the “stay-at-home” measures, participants had the possibility to progressively recover normal habits and movements outside the home. Indeed, all the shops were reopened on 18 May 2020, and all sports centres and gyms were reopened on 25 May 2020.

### 2.3. Recording and Control of Daily Physical Activity, Sedentary, and Sleep Time

To participate in the “Angel Project”, participants, after medical examinations including points 1 and 2 of the Integrative Medicine Clinic procedures (Figure S1), were requested to buy a scientifically validated commercial triaxial accelerometer, the Polar Loop 2 (Kempele, Finland) [30,31,32], to have a personal device to be continuously followed for 30 weeks and to continue to use it also after the end of their participation in the project to control and improve proper daily physical activity, sedentary and sleep characteristics. After having bought the device, an in-person appointment with the assigned researcher (i.e., a sport science specialist well-versed in physical exercise for breast cancer survivors, with more than 5 years of experience in the field of female physical activity analysis and counselling) was scheduled to explain the functioning of both the device and its connected webpage, as well as to create a personal account on it. At the end of each week, each person uploaded weekly data from the device to the webpage in order to give the assigned researcher an opportunity to analyse data and furnish, within 24 h, personalised feedback, including focus on both the positive and negative points and on and how the latter needed to be improved. The day of the data upload, before uploading, each participant recorded their morning body weight in light clothing immediately after waking up in a fasting condition and after voiding and reported it on the website. After the first period, through a new in-person appointment, each researcher furnished the operative instructions for the next period to the assigned participants. Each participant wore the device for the whole day, on the non-dominant wrist and in an adherent way. The webpage integrated the information gathered by the three-axis accelerometer with gender, age, stature, weight, and handedness of the user. As a result, qualitative, quantitative, and distributive information about both daily physical activity and sleep were obtained [30,31,32]. From the recorded data, we focused our attention on time spent in sedentary activities, and in light-, moderate-, and vigorous-intensity physical activities. Sedentary activities relate to those activities requiring an engagement ≤1.5 METs while in a sitting, reclining or lying posture and awake [33]. Light-intensity physical activities relate to those activities requiring a metabolic engagement >1.5 METs and <3 METs. Moderate-intensity physical activities relate to those activities requiring metabolic engagement ≥3 METs and ≤6 METs, while vigorous-intensity physical activities relate to those requiring a metabolic engagement >6 METs and ≤9 METs [34]. The device, combined with the webpage, furnished information about sleep characteristics. Daily nap periods were considered sedentary time, while nocturnal sleeping results are not discussed in this manuscript.

### 2.4. Dietary Habits

According to the results of basal evaluations and for the first 18 weeks, each participant received online personalized qualitative nutritional suggestions, also taking into account the symptoms and habits of the past weeks, according to the following subsequent principles: Support of organ functions, reduction of proinflammatory nutrients, reduction of nutrients eliciting increases in insulin and growth factors, and increase of nutrients stimulating the immune system. The counsellor was a nutritionist with more than 5 years of experience in the field of nutrition for breast cancer survivors. Feedback was received every 2 weeks. With a fortnight frequency, after the first 18 weeks, for 12 weeks, each participant sent information concerning the qualitative characteristics of proper nutrition and receiving the feedback about their appropriateness.

### 2.5. Live Online Physical Exercise Sessions

Live online physical exercise sessions were offered to project participants three times a week. Each workout session lasted 50 min, was conducted on the same days and hours, and was composed of a maximum of 10 women to allow exercise supervision. Twice a week, the workout included 10 min of a standing analytic warm-up, 25 min of circuit training (including two sets of seven standing and three lying down adapted calisthenic exercises), and 15 min of stretching and relaxation executed in a lying position. Once a week, the middle workout session included 10 min of a standing analytic warm-up, 25 min of standing aerobic-based exercise, and 15 min of stretching and relaxation executed in a lying position. The intensities of both calisthenics and aerobic-based exercises were assigned and controlled through the Borg 15-point RPE scale [35]. In both cases, the assigned intensity was 12–13 of the used scale. Each researcher recorded the attendance of each participant at the end of each workout. Each live online physical exercise session was conducted by a sports science specialist well-versed in physical exercise for breast cancer survivors, with more than 5 years of experience in this specific field.

### 2.6. Statistical Analysis

From 3 February 2020 to 1 June 2020, we obtained 17 weeks of continuous valid data for all of the 51 women participating in the project. The recorded weeks were gathered in four periods: 1st period (i.e., the period not including government restrictions), from 3 February 2020 to 8 March 2020; 2nd period (i.e., the first month of government restrictions), from 11 March 2020 to 7 April 2020; 3rd period (i.e., the second month of government restrictions), from 8 April 2020 to 3 May 2020; and 4th period (i.e., the first month of progressive reduction of government restrictions), from 4 May 2020 to 1 June 2020. Among the 51 recruited women, the 24 women who were able to participate in the two live online exercise sessions were placed in the E^+^ group, receiving both workouts and weekly personal counselling concerning sedentary time and physical activity. The 27 women wanting to participate but not having the ability to attend the two live online exercise sessions due to time and/or day incompatibility were placed in the E^−^ group, receiving just weekly personal advice.

The analysis of variance and chi-square test were used to verify whether subsamples differed for age, time from surgery, chemotherapy (y/n), radiation therapy (y/n), and pharmacological treatments ancillary to hormonal therapy. Basal differences of time spent in sedentary, light, moderate, and vigorous physical activities, and their variations, as well as those of body weight, according to the four periods of the study, were evaluated with linear mixed models (LMMs). As both sedentary time and physical activity could vary across time and persons, we assessed the differences among the tow exercise interventions analyzing the 1st period, as the basal (run in time), with LMMs. Mixed models increase the repeated measures precision of the estimate and provide easier handling of missing data compared to those with the ANOVA statistic. When applicable, each table contains the chosen LMM estimates and parameters, which are described at their bottom. Data, when applicable, are presented as means ± standard deviations; *p* ≤ 0.05 was considered significant. Data were analysed using the SAS 9.4 software (SAS Institute Inc., Cary, NC, USA).

## 3. Results

### 3.1. Basal Characteristics of the Sample

Table 1 shows basal characteristics of the sample, including breast cancer survivors with or without chemotherapy, radiation therapy, and pharmacological therapy to lower blood pressure and plasma lipids, in addition to hormone therapy, which are present in each person in different combinations. Table 1 also shows if subsamples (i.e., the E^−^ and E^+^ groups) differ in the reported characteristics; no differences were found, except for time spent in light and vigorous-intensity physical activities: E+ spent low time in light-intensity and more time in vigorous-intensity physical activities than E^−^. Adherence to exercise sessions in the E^+^ group was 94.37 ± 5.23%. Adherence of all participants with regard to the uploading of data was 100%.

### 3.2. Sedentary Time

Table 2 shows results concerning statistical analysis on daily sedentary time. Model A, the unconditional means model, showed that the total daily sedentary time was on average 468.25 ± 14.64 min, and the amount of variance within each person over time was 3547.98 ± 170.41 min (δ^2^_e_), whereas the amount of variation between participants, regardless of time, was 10,730.01 ± 2185.46 min (δ^2^_0_). The unconditional growth model, using the 2nd period as a reference (i.e., the first 4 weeks of government restrictions), showed that, in the 1st, 3rd, and 4th periods, participants spent less time in sedentary activities. Of the total variance, pseudo-R^2^ (R^2^_y,y1_) demonstrated that 2% could be attributable to the different periods of the study. The personal level covariate model showed that the E^−^ group spent more time in sedentary activities (49.56 ± 28.39 min) than the E^+^ group (i.e., it underlines the presence of an additive effect). Taking into account the interaction between exercise intervention and the four periods, statistical analysis did not show a multiplicative effect. Descripting the result, the E^−^ group, compared to the E^+^ group, increased its sedentary time from the 1st to the 2nd periods, while the same behaviour was shown for both subsamples during the 3rd and 4th periods (Figure S2). Of the total variance, pseudo-R^2^ (R^2^_y,y1_) demonstrated that 6% could be attributable to the interaction between time and intervention.

### 3.3. Time Spent in Light- to Vigorous-Intensity Physical Activities

Table 3 shows results concerning the statistical analysis on time spent practicing light- to vigorous-intensity physical activities. Model A, the unconditional means model, showed that daily time spent practicing light-intensity physical activities was on average 371.32 ± 11.98 min and the amount of variance within each person over time was 4411.31 ± 211.87 min (δ^2^_e_), whereas the amount of variation between participants, regardless of time, was 7071.39 ± 1463.34 min (δ^2^_0_). The unconditional growth model, using the 2nd period as a reference (i.e., the first 4 weeks of government restrictions), showed that, in the 1st, 3rd, and 4th periods, participants spent more time practicing light- to vigorous-intensity physical activities. Of the total variance, pseudo-R^2^ (R^2^_y,y1_) demonstrated that 3% could be attributable to the different periods of the study. The personal level covariate model, considering the effect of intervention and the interaction with time, showed that the E^−^ group reduced its light- to vigorous-intensity physical activities from the 1st to the 4th periods (Figure S3). Of the total variance, pseudo-R^2^ (R^2^_y,y1_) demonstrated that 7% could be attributable to the interaction between time and intervention.

### 3.4. Time Spent in Light-Intensity Physical Activities

Table 4 shows results concerning statistical analysis on time spent practicing light-intensity physical activities. Model A, the unconditional means model, showed that daily time spent practicing light-intensity physical activities was on average 310.27 ± 9.39 min and the amount of variance within each person over time was 2528.76 ± 121.45 min (δ^2^_e_), whereas the amount of variation between participants, regardless of time, was 4355.68 ± 899.26 min (δ^2^_0_). The unconditional growth model, using the 2nd period as a reference (i.e., the first 4 weeks of government restrictions), showed that, in the 1st, 3rd, and 4th periods, participants spent more time practicing light-intensity physical activities. Of the total variance, pseudo-R^2^ (R^2^_y,y1_) demonstrated that 3% could be attributable to the different periods of the study. The personal level covariate model, considering the effect of intervention and the interaction with time, shows that the E^−^ group reduced its light-intensity physical activity time from the 1st to the 4th periods (Figure S4). Of the total variance, pseudo-R^2^ (R^2^_y,y1_) demonstrated that 7% could be attributable to the interaction between time and intervention.

### 3.5. Time Spent in Moderate-Intensity Physical Activities

Table 5 shows results concerning the statistical analysis on time spent practicing moderate-intensity physical activities. Model A, the unconditional means model, showed that daily time spent practicing moderate-intensity physical activities was on average 53.09 ± 5.98 min and the amount of variance within each person over time was 716.61 ± 34.42 min (δ^2^_e_), whereas the amount of variation between participants, regardless of time, was 1781.34 ± 364.24 min (δ^2^_0_). The unconditional growth model, using the 2nd period as a reference (i.e., the first 4 weeks of government restrictions), showed that, in the 1st, 3rd, and 4th periods, participants spent more time practicing moderate-intensity physical activities. Of the total variance, pseudo-R^2^ (R^2^_y,y1_) demonstrated that 3% could be attributable to the different periods of the study. Taking into account the interaction between exercise intervention and the four periods, statistical analysis did not show a multiplicative but just a time effect. Describing the result, the E^−^ group, compared to the E^+^ group, reduced its moderate-intensity physical activities from the 1st to the 2nd periods, while the same behaviour was shown for both subsamples during the 3rd and 4th periods (Figure S5). Of the total variance, pseudo-R^2^ (R^2^_y,y1_) demonstrated that 4% could be attributable to the interaction between time and intervention.

### 3.6. Time Spent in Vigorous-Intensity Physical Activities

Table 6 shows results concerning the statistical analysis on time spent practicing vigorous-intensity physical activities. Model A, the unconditional means model, showed that daily time spent practicing vigorous-intensity physical activities was on average 7.96 ± 1.34 min and the amount of variance within each person over time was 93.03 ± 4.46 min (δ^2^_e_), whereas the amount of variation between participants, regardless of time, was 86.49 ± 18.33 min (δ^2^_0_). The unconditional growth model, using the 2nd period as a reference (i.e., the first 4 weeks of government restrictions), showed that, in the 1st, 3rd, and 4th periods, participants spent more time practicing vigorous-intensity physical activities. Of the total variance, pseudo-R^2^ (R^2^_y,y1_) demonstrated that 2% could be attributable to the different periods of the study. Taking into account the interaction between exercise intervention and the four periods, statistical analysis did not show a multiplicative but just a time effect. Describing the result, the E^−^ group, compared to the E^+^ group, reduced its vigorous-intensity physical activities from the 1st to the 2nd periods, while the same behaviour was shown for both subsamples during the 3rd and 4th periods (Figure S6). Of the total variance, pseudo-R^2^ (R^2^_y,y1_) demonstrated that 6% could be attributable to the interaction between time and intervention.

### 3.7. Body Weight

**Figure 1:**
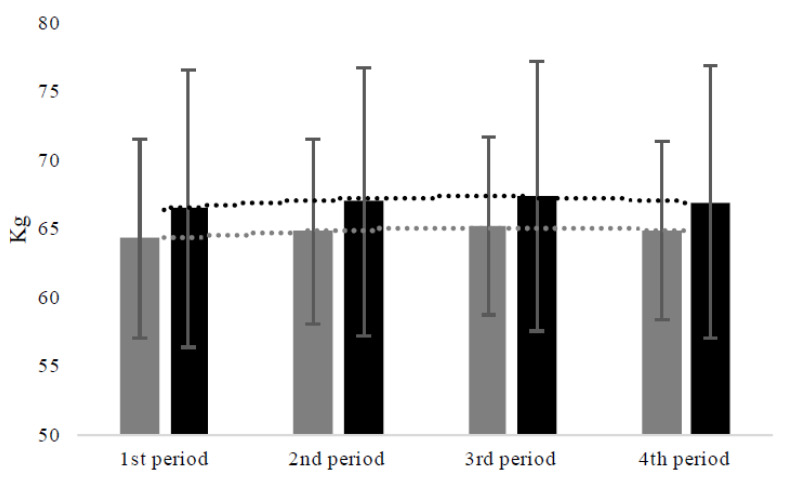
Variation of body weight according to the time of the study and type of intervention, with just a significant time effect, according to the linear mixed models analysis. Note: Grey columns, non-exercising women; black columns, exercising women; 1st period (i.e., the period not including government restrictions), from 3 February 2020 to 8 March 2020; 2nd period (i.e., the first month of government restrictions), from 11 March 2020 to 7 April 2020; 3rd period (i.e., the second month of government restrictions), from 8 April 2020 to 3 May 2020; 4th period (i.e., the first month of progressive reduction of government restrictions), from 4 May 2020 to 1 June 2020.

When the same statistical analysis was repeated for body weight, no significant effects were shown for intervention and for its interaction with time. On the contrary, a significant time effect was shown (F_(3,147)_ = 27.62; *p* < 0.001) (Figure 1). Specifically, using the 2nd period as a reference (i.e., the first 4 weeks of government restrictions), our data showed that body weight significantly increased from the 1st (Est. = −0.33; St. Error = 0.09; df = 150; t = −3.72; *p* < 0.001) to 3rd (Est. = 0.25; St. Error = 0.08; df = 150; t = 2.87; *p* < 0.004) periods and then declined in the 4th period (i.e., the first month of progressive reduction of government restrictions) (Est. = −0.28; St. Error = 0.12; df = 150; t = −2.25; *p* = 0.02).

## 4. Discussion

Concerning sedentary time and physical activity, the important result of our study is that, notwithstanding the presence of weekly personal advice and the use of technology, the absence of participation in a supervised physical exercise programme did not allow for effective improvement in the daily movement of breast cancer survivors in the presence of a situation simultaneously limiting geographic mobility and socialization, such as the COVID-19 emergency. Our results also showed that the use of technology allows the promotion of “self-knowledge, and health, through numbers” [19], as each participant had the possibility, to see, through the webpage, proper sedentary time and physical activity trends; through the activity tracker, the instantaneous amount of daily physical activity; and had the possibility to be alerted when 1 h of continuous sedentary time was reached. These results could be explained through those of Wu et al. [18], even though their study was not conducted in a situation such as that created by the COVID-19 emergency. Indeed, even though their participants reported that the activity tracker and its accompanying app functioned as a motivational tool and created more awareness of physical activity and sedentary behaviour, they underlined that tailored and personalized advice were particularly important, as well as the role of the physiotherapist giving them every 2 weeks. Indeed, all the women had undergone cancer treatment and experienced disease- and treatment-related side effects, as well as fatigue and a decreased physical fitness level, and according to participants, it was sometimes frustrating to read messages generated by a system that did not consider the side effects of the treatment. Translating these results in our case, including women participating in online supervised exercise sessions and women who did not (all receiving personalized advice every week), we probably have a similar situation—the presence of limitations concerning geographic mobility and socialization, creating a negative self-reinforcing loop including fear, anxiety, sedentary time, and treatment-related side effects, such as fatigue and a decreased physical fitness level, does not allow properly applying the tailored advice, stagnating the person in a poor psychophysical condition that is characterized by the increase of sedentary time and the decrease of daily physical activity [12,24,27]. On the contrary, online supervised exercise sessions could be used to safely maintain/improve the daily physical activity level of breast cancer survivors in the presence of personal restrictions, since thanks to its characteristics, physical exercise properly reduces/prevents some negative side effects of treatment, such as fatigue and pain, negatively affecting daily movements, and promotes psychological health, which is undoubtedly linked with physical health and daily physical activity [12,19,36]. Nevertheless, statistical analysis did not show a significant interaction of intervention with time, both for moderate- and vigorous-intensity physical activities, although the E^+^ group had a mean adherence to exercise sessions of 94.37 ± 5.23%. This stimulates two hypotheses. The first is that, while the E^+^ group partially replaced daily moderate- to vigorous-intensity spontaneous physical activities with live online workouts, being more functional for health, due to their characteristics (i.e., proper total and continuous duration, proper modulation of both intensity and recovery, and proper activity selection), the E^−^ group did not and tried to maintain its daily routines. The second hypothesis is that the activity tracker simply underestimated some of the physical exercises that were performed on the spot, recognizing it as light-intensity physical activity or sedentary time [37]. Indeed, when we focused the attention on the analysis of the trend of time spent on light- to vigorous-intensity physical activities and on that of light-intensity physical activities, we observed the presence of a multiplicative effect, with the E^+^ group showing a better trend. Therefore, it is conceivable to speculate that, in the presence of personal limitations, breast cancer survivors participating in online supervised exercise sessions, physical activity monitoring and counselling programmes, as presented, benefit from a tailored theoretical and practical intervention positively affecting their daily physical activity. On the contrary, when the online supervised exercise sessions are not present, breast cancer survivors benefit from just a theoretical intervention that, according to our results, is not enough to support proper daily physical activity recomposition (i.e., sedentary time reduction with a contemporaneous increase of light-, moderate- and/or vigorous-intensity physical activities). We are also in accordance with Newton et al. [36] reporting that, in the era of COVID-19, it is necessary that exercise oncology programmes adapt to the changing environment, as patients with cancer and survivors risk to regress to a sedentary lifestyle, resulting in a decline of health and their quality of life, particularly those undergoing treatment or suffering adverse effects of treatment. To do this, according to Newton et al. [36], the key elements are online exercise led by an exercise professional that has to be able to create a tailored lesson and a personal interaction. Interpreting our results, the willful condition of breast cancer survivors is particularly important since, according to Gardner et al. [38], if self-control is not diminished, habit formation alone may not be sufficient for the maintenance of behaviour change. The statement by Gardner et al. [38] is also supported by the observations of the unconditional growth models of sedentary time and physical activity variables, notwithstanding the increase of sedentary time and the decrease of light-, moderate-, and vigorous-intensity physical activities from the 1st to 2nd periods. Moving on from the 2nd period, it is possible to observe, in the E^−^ group, the inversion of the trends reaching the same values of the 1st period (Table 2, Table 3, Table 4, Table 5 and Table 6, Figures S2–S6). Therefore, if habit alone had been sufficient for the maintenance of behaviour change, we would have had to observe a stagnation of the variables and not a return to the starting point.

When body weight was analysed, just a time effect was found, showing its increase from the 1st to the 3rd periods and then its decrease, with a mean variation from one period to another widely lower than 1 kg. The absence of a significant multiplicative effect in body weight, notwithstanding the presence of a significant additive effect in sedentary time and multiplicative effect in both physical activity and physical exercise participation, allows us to hypothesize that the subsamples differently managed quantity of food. Indeed, the presence of different trends in sedentary time and physical activity, with a same trend in body weight, allowed speculating that the E^−^ group probably adopted a more quantitatively restricted diet to compensate for inadequate daily movements and the inability to better address it, notwithstanding personal advice, without live online exercise sessions. Unfortunately, the absence of objective data concerning the nutritional habits of study participants, including both qualitative and quantitative information, does not allow going beyond this hypothesis, as we furnished fortnight personal qualitative nutritional counselling, without knowing how effectively they applied them and with what quantities.

Study limitations included: (i) The absence of objective data concerning the nutritional habits of study participants, including both qualitative and quantitative information; (ii) with regard to sample characteristics, indeed, we had a sample of breast cancer survivors having the possibility to buy an activity tracker, even if it is a low-cost device, to use it in conjunction with its web app to have an Internet connection and both ability and possibility to routinely connect for workout and/or nutritional suggestions. This implies that our results are not generalizable to the whole population but are applicable just to a similar population, probably with a middle-high socioeconomic status and probably under the “healthy worker effect” [39], as the volition to participate in the project means that participants have the volition to improve their daily physical activity; (iii) the absence of data concerning psychological fields, which certainly could close the circle and better illustrate the trend of all components of behaviour, allowing us to better identify the causes, mediators, and correlates of the trend of each area; (iv) the interaction between on spot exercises, including callisthenic exercises, with just a wrist accelerometer that represents a partial study limitation, as it is possible to obtain an underestimation of the intensity due to body position, notwithstanding that the body was moderately to vigorously engaged. Lastly, another study limitation concerns the low effect size of our results, expressed as (R^2^_y,y1_), meaning that the models considered could explain only a little percentage of the total variance. Therefore, the presence of day-to-day data, coming from 17 consecutive weeks, concerning sedentary time and physical activity variables and the COVID-19 emergency represented the strength of the study. Indeed, the latter, through the government restrictions, allowed us to observe the personal response to, until now, a unique situation in the technological era, furnishing the possibility to translate them in a similar population (i.e., cancer survivors requesting the same pharmacological treatments) and in a similar situation (i.e., pandemic emergency and personal restrictions due to immune deficiency not allowing social activities).

## 5. Conclusions

Our results suggest that the use of an activity tracker, its accompanying app, and the reception of weekly tailored advice concerning the improvement of sedentary time and physical activity are not enough to elicit proper physical activity recomposition in breast cancer survivors in the COVID-19 era. On the contrary, using them in addition to online supervised physical exercise sessions seems able to counteract the negative effects of COVID-19 personal restrictions on sedentary time and physical activity. Therefore, the COVID-19 pandemic emergency and its related government restrictions have been shown to not negatively influence the sedentary time and daily physical activity of breast cancer survivors prone to change, recovering their behaviour when restrictions were reduced. According to our opinion, our results could be translated into situations similar to that of COVID-19, including patients with breast cancer needing particular attention and confinement, in order to optimize health through movement, remembering that, in the field of physical activity, merging technology with the live and tailored approach seems the optimal combination to favor health through movement, as it furnishes the possibility to move without discomfort and, as a consequence, to continue to move, to increase the fitness level and explore new opportunities of movement and health.

## Figures and Tables

**Table 1:** Basal characteristics of the sample and basal differences among subsamples.

	N = 51	E^−^ (*n* = 27)	E^+^ (*n* = 24)	E^−^ vs. E^+^ *p*
Age (years)	50.98 ± 6.17	50.62 ± 3.71	51.37 ± 8.18	0.67
Time from surgery (months)	13.68 ± 7.03	14.14 ± 6.72	13.16 ± 7.46	0.62
Chemotherapy (y/n)	22/29	12/15	10/14	0.49
Radiation therapy (y/n)	33/18	18/9	15/9	0.28
Blood pressure-lowering drugs (y/n)	37/14	6/21	8/16	0.28
Lipid-lowering drugs (y/n)	29/22	9/18	7/17	0.49
Sedentary time (min)	457.62 ± 101.36	465.67 ± 97.36	448.56 ± 105.36	0.53
Light-intensity physical activities (min)	327.42 ± 90.00	351.77 ± 87.77	300.01 ± 84.77	0.02
Moderate-intensity physical activities (min)	62.15 ± 41.96	60.53 ± 37.96	63.98 ± 46.15	0.81
Vigorous-intensity physical activities (min)	10.51 ± 16.81	6.08 ± 10.78	15.48 ± 20.62	0.04

Statistical significances concerning age and time from surgery are inherent to the analysis of variance. Statistical significances concerning chemotherapy, radiation therapy, blood pressure-lowering drugs, and lipid-lowering drugs are inherent to the Chi-square test, while statistical significances concerning other variables are inherent to the LMMs.

**Table 2:** Mixed model analyses of variations in sedentary time.

			Model A Unconditional Means Model	Model B Unconditional Growth Model	Model C Personal Level Covariate
Initial status	Intercept	γ_00_	468.25 ± 14.64 ***	482.50 ± 14.31 ***	456.18 ± 20.66 ***
	Intervention	γ_01_			49.56 ± 28.39 *
Rate of change	Intercept (time)	γ_10_-1		−24.90 ± 5.60 ***	−8.18 ± 8.03
		γ_10_-2		Reference	Reference
		γ_10_-3		−10.92 ± 5.60 *	−17.88 ± 8.05 *
		γ_10_-4		−26.59 ± 7.56 ***	−35.05 ± 10.78 **
Interaction	Time * intervention				
		γ_11_-1			−31.53 ± 11.04 **
		γ_11_-2			Reference
		γ_11_-3			13.09 ± 11.06
		γ_11_-4			15.89 ± 14.82
Level 1	Within-person	δ^2^_e_	3547.98 ± 170.41 ***	2417.51 ± 119.84 ***	2415.17 ± 119.91 ***
Level 2	In initial status	δ^2^_0_	10,730.01 ± 2185.46 ***	9582.94 ± 2015.14 ***	9730.47 ± 2064.95 ***
	In rate of change	δ^2^_1_		35.68 ± 8.09 **	31.89 ± 7.39 ***
	Covariance	δ_01_		−81.03 ± 91.87	−99.58 ± 89.64
		ρ	0.75		
		R^2^_y,y1_		0.02	0.06
		R^2^_e_			0.30
		R^2^_0_			0.01
		R^2^_1_			0.13
		AIC	10,309	10,063	10,025
		BIC	10,313	10,071	10,032

Note: * 0.05 < *p*< 0.01, ** 0.01 ≤ *p* < 0.001, *** *p* ≤ 0.001. Abbreviations: γ_00_ = intercept of the average trajectory; γ_01_ = intercept of the intervention trajectory; γ_10_-1 = intercept time effect of the trajectory for the run-in period; γ_10_-2 = reference time, 2nd period; γ_10_-3 = intercept of the trajectory for the 3rd period; γ_10_-4 = intercept of the trajectory for the 4th period; γ_11_-1 = slope of the trajectory for the interaction between intervention and run-in phase/period; γ_11_-2 = reference; γ_11_-3 = slope of the trajectory for the interaction between intervention and 3rd phase/period; γ_11_-4 = slope of the trajectory for the interaction between intervention and 4th phase/period; δ^2^_e_ = within-person variance components; δ^2^_0_ = in initial status variance components; δ^2^_1_ = in rate of change variance components; δ_01_ = covariance estimate; ρ = intraclass coefficient correlation; R^2^_y,y1_ = percentage of total variability associated linearly with time; R^2^_e_ = pseudo-R^2^ statistic assesses the proportion of within-person variation “explained by time”; R^2^_0_ = pseudo-R^2^ statistic assesses the percentage variation in initial status; R^2^_1_ = pseudo-R^2^ statistic assesses the percentage variation in rate of change; AIC = Akaike information criterion; BIC = Bayesian information criterion.

**Table 3:** Mixed model analyses of variation of light- to vigorous-intensity physical activities.

			Model A Unconditional Means Model	Model B Unconditional Growth Model	Model C Personal Level Covariate
Initial status	Intercept	γ_00_	371.32 ± 11.98 ***	341.75 ± 12.23 ***	338.78 ± 17.97 ***
	Intervention	γ_01_			5.86 ± 24.70
Rate of change	Intercept (time)	γ_10_-1		54.13 ± 5.90 ***	33.30 ± 11.63 **
		γ_10_-2		Reference	Reference
		γ_10_-3		24.69 ± 5.88 ***	−16.89 ± 11.61
		γ_10_-4		54.78 ± 8.02 ***	−35.86 ± 15.73 *
Interaction	Time * intervention				
		γ_11_-1			19.67 ± 9.53 *
		γ_11_-2			Reference
		γ_11_-3			5.27 ± 9.94
		γ_11_-4			47.55 ± 11.76 ***
Level 1	Within-person	δ^2^_e_	4411.31 ± 211.87 ***	2568.43 ± 127.38 ***	2561.44 ± 127.20 ***
Level 2	In initial status	δ^2^_0_	7071.39 ± 1463.34 ***	10,265 ± 2159.66 ***	9984.46 ± 2120.10 ***
	In rate of change	δ^2^_1_		43.94 ± 9.85 ***	39.55 ± 9.01 ***
	Covariance	δ_01_		−368.56 ± 118.64 **	−325.05 ± 110.97 **
		ρ	0.64		
		R^2^_y,y1_		0.03	0.07
		R^2^_e_			0.37
		R^2^_0_			0.04
		R^2^_1_			0.10
		AIC	10,478	10,100	10,069
		BIC	10,482	10,115	10,077

Note: * 0.05 < *p* < 0.01, ** 0.01 ≤ *p* < 0.001, *** *p* ≤ 0.001. Abbreviations: γ_00_ = intercept of the average trajectory; γ_01_ = intercept of the intervention trajectory; γ_10_-1 = intercept time effect of the trajectory for the run-in period; γ_10_-2 = reference time, 2nd period; γ_10_-3 = intercept of the trajectory for the 3rd period; γ_10_-4 = intercept of the trajectory for the 4th period; γ_11_-1 = slope of the trajectory for the interaction between intervention and run-in phase/period; γ_11_-2 = reference; γ_11_-3 = slope of the trajectory for the interaction between intervention and 3rd phase/period; γ_11_-4 = slope of the trajectory for the interaction between intervention and 4th phase/period; δ^2^_e_ = within-person variance components; δ^2^_0_ = in initial status variance components; δ^2^_1_ = in rate of change variance components; δ_01_ = covariance estimate; ρ = intraclass coefficient correlation; R^2^_y,y1_ = percentage of total variability associated linearly with time; R^2^_e_ = pseudo-R^2^ statistic assesses the proportion of within-person variation “explained by time”; R^2^_0_ = pseudo-R^2^ statistic assesses the percentage variation in initial status; R^2^_1_ = pseudo-R^2^ statistic assesses the percentage variation in rate of change; AIC = Akaike information criterion; BIC = Bayesian information criterion.

**Table 4:** Mixed model analyses of variations in light-intensity physical activities.

			Model A Unconditional Means Model	Model B Unconditional Growth Model	Model C Personal Level Covariate
Initial status	Intercept	γ_00_	310.27 ± 9.39 ***	292.06 ± 9.64 ***	279.85 ± 13.97 ***
	Intervention	γ_01_			23.21 ± 19.20
Rate of change	Intercept (time)	γ_10_-1		34.70 ± 4.58 ***	20.05 ± 6.51 **
		γ_10_-2		Reference	Reference
		γ_10_-3		13.72 ± 4.61 **	20.63 ± 6.58 **
		γ_10_-4		31.81 ± 6.10 ***	45.81 ± 8.61 ***
Interaction	Time * intervention				
		γ_11_-1			27.76 ± 8.95 **
		γ_11_-2			Reference
		γ_11_-3			−13.11 ± 9.04
		γ_11_-4			−26.57 ± 11.83 *
Level 1	Within-person	δ^2^_e_	2528.76 ± 121.45 ***	1740.58 ± 86.28 ***	1736.73 ± 86.20 ***
Level 2	In initial status	δ^2^_0_	4355.68 ± 899.26 ***	6965.27 ± 1460.77 ***	6331.01 ± 1345.51 ***
	In rate of change	δ^2^_1_		19.84 ± 4.67 ***	16.82 ± 4.08 ***
	Covariance	δ_01_		−235.36 ± 69.32 ***	−188.39 ± 60.84 **
		ρ	0.63		
		R^2^_y,y1_		0.03	0.07
		R^2^_e_			0.28
		R^2^_0_			0.11
		R^2^_1_			0.17
		AIC	9971	9727	9688
		BIC	9975	9735	9696

Note: * 0.05 < *p* < 0.01, ** 0.01 ≤ *p* < 0.001, *** *p* ≤ 0.001. Abbreviations: γ_00_ = intercept of the average trajectory; γ_01_ = intercept of the intervention trajectory; γ_10_-1 = intercept time effect of the trajectory for the run-in period; γ_10_-2 = reference time, 2nd period; γ_10_-3 = intercept of the trajectory for the 3rd period; γ_10_-4 = intercept of the trajectory for the 4th period; γ_11_-1 = slope of the trajectory for the interaction between intervention and run-in phase/period; γ_11_-2 = reference; γ_11_-3 = slope of the trajectory for the interaction between intervention and 3rd phase/period; γ_11_-4 = slope of the trajectory for the interaction between intervention and 4th phase/period; δ^2^_e_ = within-person variance components; δ^2^_0_ = in initial status variance components; δ^2^_1_ = in rate of change variance components; δ_01_ = covariance estimate; ρ = intraclass coefficient correlation; R^2^_y,y1_ = percentage of total variability associated linearly with time; R^2^_e_ = pseudo-R^2^ statistic assesses the proportion of within-person variation “explained by time”; R^2^_0_ = pseudo-R^2^ statistic assesses the percentage variation in initial status; R^2^_1_ = pseudo-R^2^ statistic assesses the percentage variation in rate of change; AIC = Akaike information criterion; BIC = Bayesian information criterion.

**Table 5:** Mixed model analyses of variations in moderate-intensity physical activities.

			Model A Unconditional Means Model	Model B Unconditional Growth Model	Model C Personal Level Covariate
Initial status	Intercept	γ_00_	53.09 ± 5.98 ***	47.99 ± 5.36 ***	55.43 ± 7.84 ***
	Intervention	γ_01_			−14.28 ± 10.77
Rate of change	Intercept (time)	γ_10_-1		14.74 ± 2.38 ***	8.99 ± 3.45 *
		γ_10_-2		Reference	Reference
		γ_10_-3		9.62 ± 2.33 ***	11.55 ± 3.38 ***
		γ_10_-4		20.50 ± 3.31 ***	23.29 ± 4.80 ***
Interaction	Time * intervention				
		γ_11_-1			10.91 ± 4.74 *
		γ_11_-2			Reference
		γ_11_-3			−3.71 ± 4.65
		γ_11_-4			−5.39 ± 6.59
Level 1	Within-person	δ^2^_e_	716.61 ± 34.42 ***	345.29 ± 17.14 ***	344.68 ± 17.13 ***
Level 2	In initial status	δ^2^_0_	1781.34 ± 364.24 ***	1200.33 ± 254.36 ***	1223.10 ± 261.47 ***
	In rate of change	δ^2^_1_		11.36 ± 2.43 ***	11.09 ± 2.38 ***
	Covariance	δ_01_		−12.05 ± 17.93	−13.31 ± 17.99
		Ρ	0.71		
		R^2^_y,y1_		0.03	0.04
		R^2^_e_			0.48
		R^2^_0_			0.03
		R^2^_1_			0.01
		AIC	8833	8316	8289
		BIC	8837	8323	8297

Note: * 0.05 < *p* < 0.01, *** *p* ≤ 0.001. Abbreviations: γ_00_ = intercept of the average trajectory; γ_01_ = intercept of the intervention trajectory; γ_10_-1 = intercept time effect of the trajectory for the run-in period; γ_10_-2 = reference time, 2nd period; γ_10_-3 = intercept of the trajectory for the 3rd period; γ_10_-4 = intercept of the trajectory for the 4th period; γ_11_-1 = slope of the trajectory for the interaction between intervention and run-in phase/period; γ_11_-2 = reference; γ_11_-3 = slope of the trajectory for the interaction between intervention and 3rd phase/period; γ_11_-4 = slope of the trajectory for the interaction between intervention and 4th phase/period; δ^2^_e_ = within-person variance components; δ^2^_0_ = in initial status variance components; δ^2^_1_ = in rate of change variance components; δ_01_ = covariance estimate; ρ = intraclass coefficient correlation; R^2^_y,y1_ = percentage of total variability associated linearly with time; R^2^_e_ = pseudo-R^2^ statistic assesses the proportion of within-person variation “explained by time”; R^2^_0_ = pseudo-R^2^ statistic assesses the percentage variation in initial status; R^2^_1_ = pseudo-R^2^ statistic assesses the percentage variation in rate of change; AIC = Akaike information criterion; BIC = Bayesian information criterion.

**Table 6:** Mixed model analyses of variations in vigorous-intensity physical activities.

			Model A Unconditional Means Model	Model B Unconditional Growth Model	Model C Personal Level Covariate
Initial status	Intercept	γ_00_	7.96 ± 1.34 ***	5.80 ± 1.40 ***	7.36 ± 1.98 ***
	Intervention	γ_01_			−2.79 ± 2.73
Rate of change	Intercept (time)	γ_10_-1		4.00 ± 0.90 ***	6.98 ± 1.30 *
		γ_10_-2		Reference	Reference
		γ_10_-3		1.97 ± 0.91 *	1.87 ± 1.31
		γ_10_-4		3.53 ± 1.20 **	5.37 ± 1.73 **
Interaction	Time * intervention				
		γ_11_-1			5.62 ± 1.79 **
		γ_11_-2			Reference
		γ_11_-3			0.17 ± 1.80
		γ_11_-4			−3.50 ± 2.38
Level 1	Within-person	δ^2^_e_	93.03 ± 4.46 ***	69.34 ± 3.44 ***	68.21 ± 3.39 ***
Level 2	In initial status	δ^2^_0_	86.49 ± 18.33 ***	142.82 ± 31.53 ***	128.28 ± 28.90 **
	In rate of change	δ^2^_1_		0.73 ± 0.17 ***	0.71 ± 0.17 ***
	Covariance	δ_01_		−6.36 ± 1.99 **	−5.65 ± 1.87
		ρ	0.48		
		R^2^_y,y1_		0.02	0.06
		R^2^_e_			0.24
		R^2^_0_			0.11
		R^2^_1_			0.03
		AIC	6913	6747	6713
		BIC	6917	6755	6721

Note: * 0.05 < *p* < 0.01, ** 0.01 ≤ *p* < 0.001, *** *p* ≤ 0.001. Abbreviations: γ_00_ = intercept of the average trajectory; γ_01_ = intercept of the intervention trajectory; γ_10_-1 = intercept time effect of the trajectory for the run-in period; γ_10_-2 = reference time, 2nd period; γ_10_-3 = intercept of the trajectory for the 3rd period; γ_10_-4 = intercept of the trajectory for the 4th period; γ_11_-1 = slope of the trajectory for the interaction between intervention and run-in phase/period; γ_11_-2 = reference; γ_11_-3 = slope of the trajectory for the interaction between intervention and 3rd phase/period; γ_11_-4 = slope of the trajectory for the interaction between intervention and 4th phase/period; δ^2^_e_ = within-person variance components; δ^2^_0_ = in initial status variance components; δ^2^_1_ = in rate of change variance components; δ_01_ = covariance estimate; ρ = intraclass coefficient correlation; R^2^_y,y1_ = percentage of total variability associated linearly with time; R^2^_e_ = pseudo-R^2^ statistic assesses the proportion of within-person variation “explained by time”; R^2^_0_ = pseudo-R^2^ statistic assesses the percentage variation in initial status; R^2^_1_ = pseudo-R^2^ statistic assesses the percentage variation in rate of change; AIC = Akaike information criterion; BIC = Bayesian information criterion.

## Data Availability

The data presented in this study are available on request from the corresponding author.

## Supplementary Materials

The following are available online at https://www.mdpi.com/article/10.3390/jfmk6020050/s1.
