# Supplementary material for: Effects of Activity Tracker-Based Counselling and Live-Web Exercise on Breast Cancer Survivors during Italy COVID-19 Lockdown
Source: J Funct Morphol Kinesiol. 2021 Jun 9;6(2):50. doi: 10.3390/jfmk6020050 (PMC8293416; doi:10.3390/jfmk6020050)
Supplement: Supplementary file 1 [file jfmk-06-00050-s001.zip › Supplementary figures/Figure S6.pdf]

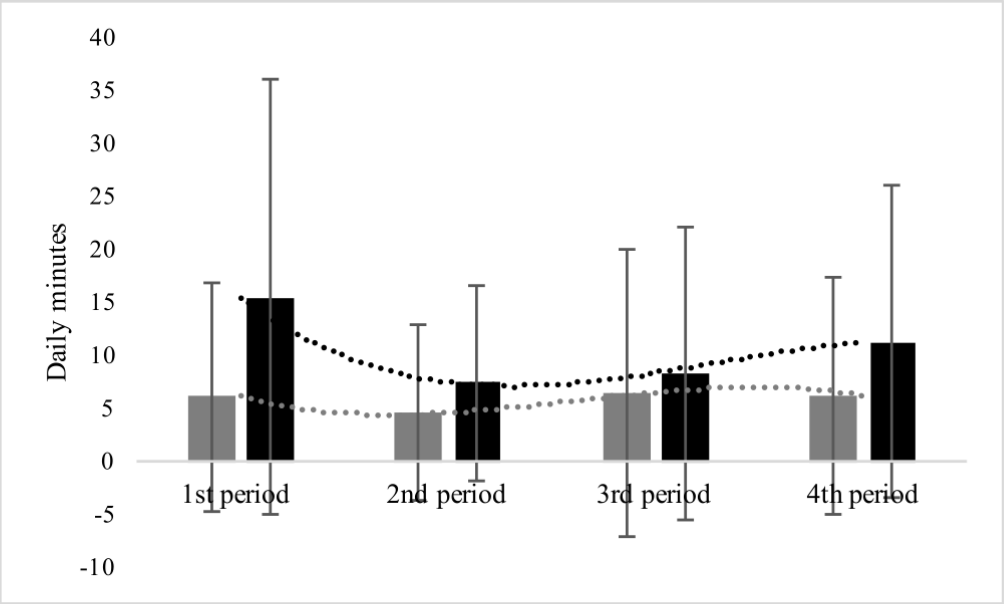

Figure S6. *Variation of daily of daily time spent in vigorous-intensity physical activities according to time of the study and type of intervention*

Note: grey columns, non-exercising women; black columns, exercising women; 1st period (i.e., the period not including government restrictions), from 3 February 2020 to 8 March 2020; 2nd period (i.e., the first month of government restrictions), from 11 March 2020 to 7 April 2020; 3rd period (i.e., the second month of government restrictions), from 8 April 2020 to 3 May 2020; 4th period (i.e., the first month of progressive reduction of government restrictions), from 4 May 2020 to 1 June 2020. See Table 6 for data concerning statistical significance.
