# Supplementary figures and images for: Effects of Activity Tracker-Based Counselling and Live-Web Exercise on Breast Cancer Survivors during Italy COVID-19 Lockdown
Source: J Funct Morphol Kinesiol. 2021 Jun 9;6(2):50. doi: 10.3390/jfmk6020050 (PMC8293416; doi:10.3390/jfmk6020050)

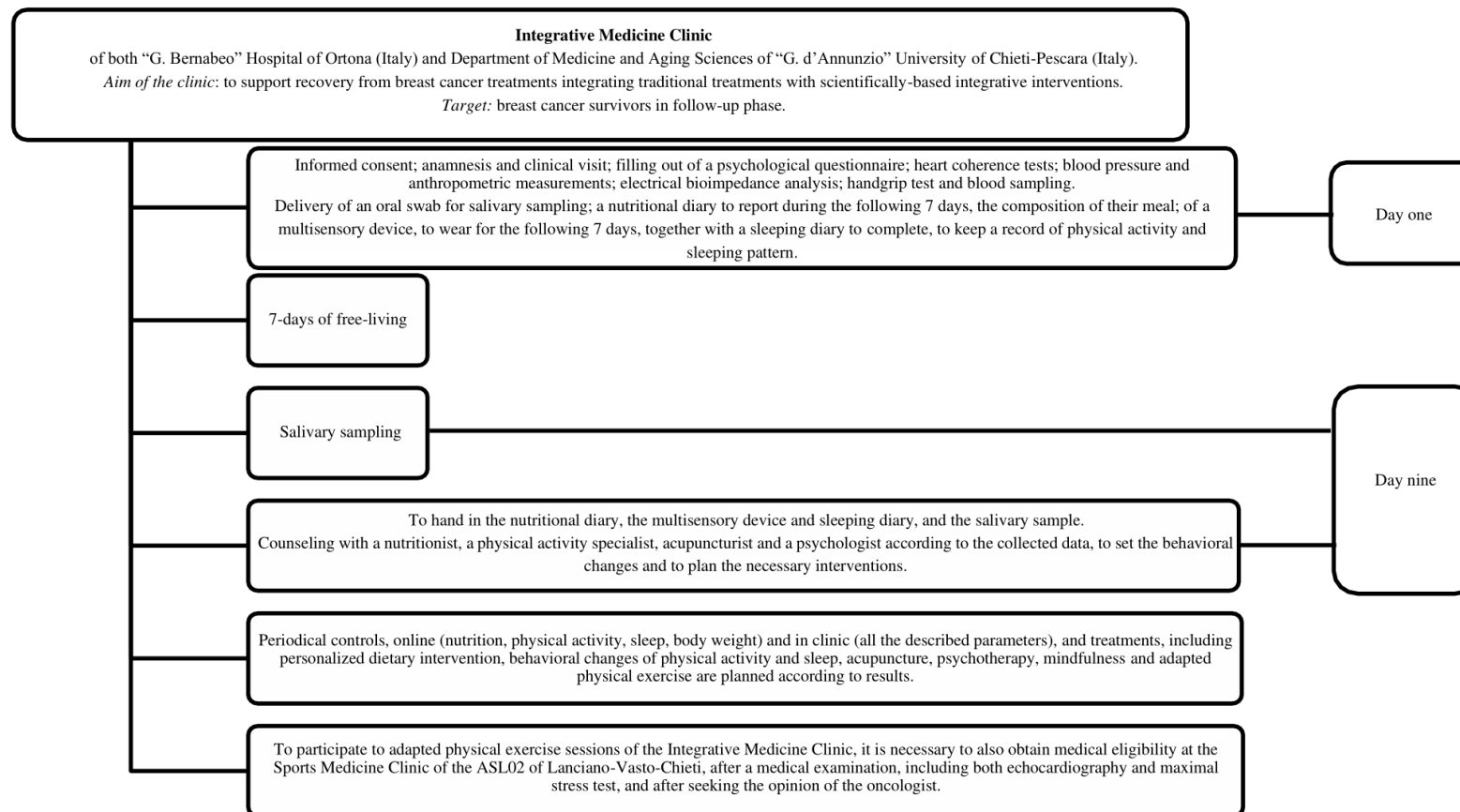

Figure S1. *Study design*

Supplement: Supplementary file 1 [file jfmk-06-00050-s001.zip › Supplementary figures/Figure S1.pdf]
